# A Linguistic–Sensorimotor Model of the Basic‐Level Advantage in Category Verification

**DOI:** 10.1111/cogs.70025

**Published:** 2024-12-23

**Authors:** Cai Wingfield, Rens van Hoef, Louise Connell

**Affiliations:** ^1^ Department of Psychology Lancaster University; ^2^ Department of Psychology Maynooth University

**Keywords:** Categories, Basic level, Concepts, Linguistic distributional, Sensorimotor

## Abstract

People are generally more accurate at categorizing objects at the basic level (e.g., *dog*) than at more general, superordinate categories (e.g., *animal*). Recent research has suggested that this basic‐level advantage emerges from the linguistic‐distributional and sensorimotor relationship between a category concept and object concept, but the proposed mechanisms have not been subject to a formal computational test. In this paper, we present a computational model of category verification that allows linguistic distributional information and sensorimotor experience to interact in a grounded implementation of a full‐size adult conceptual system. In simulations across multiple datasets, we demonstrate that the model performs the task of category verification at a level comparable to human participants, and—critically—that its operation naturally gives rise to the basic‐level‐advantage phenomenon. That is, concepts are easier to categorize when there is a high degree of overlap in sensorimotor experience and/or linguistic distributional knowledge between category and member concepts, and the basic‐level advantage emerges as an overall behavioral artifact of this linguistic and sensorimotor overlap. Findings support the linguistic–sensorimotor preparation account of the basic‐level advantage and, more broadly, linguistic–sensorimotor theories of the conceptual system.

## 1 Introduction

Categorization—carving up the objects we perceive in the world into meaningful groups—is a fundamental cognitive action (Harnad, 2005; Rosch, 1978). Importantly, any given object (e.g., a small, feathered creature) may simultaneously be categorized in multiple classes (e.g., *goldfinch*, *finch*, *bird*, *animal*, *living thing*, etc.) that can often be arranged to form a structure resembling a taxonomy: an inclusive hierarchy ranging from highly specific to highly general. While an object may be categorized at any taxonomic level, research has shown a distinct processing advantage for categories found at the intermediate “basic” level (e.g., *bird*), whereby basic‐level category names are acquired earlier by children (Brown, 1958; Rosch, Mervis, Gray, Johnson, & Boyes‐Braem, 1976), and produced faster and more frequently in object naming (Jolicoeur, Gluck, & Kosslyn, 1984; Rosch et al., 1976), than superordinate (more general: e.g., *animal*) or subordinate (more specific; e.g., *goldfinch*) category names. The strongest evidence for the basic‐level advantage in categorization comes from label → picture category verification tasks. In such tasks, participants are shown category labels followed by object pictures, and asked to judge whether the depicted object belongs to the preceding category in a two‐alternative forced choice judgment (e.g., yes/no or member/nonmember). Using this paradigm, a multitude of studies has confirmed that participants are generally faster and more accurate to verify category membership when the pictured item (e.g., picture of a goldfinch) is preceded by a basic‐level label (e.g., *bird*), compared to a superordinate label (e.g., *animal*) or in most circumstances a subordinate label (e.g., *goldfinch*: Johnson & Mervis, 1997; Jolicoeur et al., 1984; Murphy & Brownell, 1985; Rosch et al., 1976; Tanaka & Taylor, 1991; van Hoef, Connell, & Lynott, 2023).

Traditional explanations for the basic‐level advantage effect either assume the basic level is the preferred entry level into a taxonomically organized semantic memory (Jolicoeur et al., 1984), or is the category that is most optimally differentiated from others by its semantic features (e.g., Murphy & Brownell, 1985). While their process models differ, such explanations of the basic‐level advantage typically assume concepts comprise discrete, binary features (e.g., *has wings*, *can fly*). As such, they are in line with broader theories of categorization that assume concepts form categories based on their feature similarity (e.g., family resemblance theory: Rosch & Mervis, 1975; Wittgenstein, 1953; or exemplar theory: Medin & Schaffer, 1978; Nosofsky, 1986). Feature‐based representations of concepts are intuitive (e.g., it can be easily understood that a *bird* usually *has wings*, but a *dog* does not) and can include rich semantic detail, but there are several accompanying shortcomings that have made them the subject of criticism. Semantic features are typically elicited in property‐generation tasks and are thus limited to those aspects of conceptual representation that can be easily verbalized (Banks & Connell, 2022b; Reilly et al., 2024). Even when verbalized, feature‐based representations can lack consistent grounding (e.g., *clock* is *used to tell time*) and can be difficult to extend into abstract and non–noun concepts (Wingfield & Connell, 2022a; see also Yee et al., 2013). Moreover, features (e.g., “*has wings*”) are often complex concepts in their own right, which in turn should have their own set of semantic features (e.g., what are the features of *wings*?), which in turn should have features of their own (e.g., if *feathers* is a feature of *wings*, what are the features of *feathers*?). The resulting combinatorial explosion limits the explanatory value of theories that rely on such representations (Binder et al., 2016). More specifically, it is not apparent how feature‐similarity can underpin categorization when the meaning of particular features only becomes apparent once the category is known (e.g., “*is large*” has a different meaning for *cups* than it does for *pianos;* Medin & Shoben, 1988). Indeed, a recent comparison of semantic similarity metrics found that feature‐similarity was overall the weakest predictor of human similarity judgments^1^ (Wingfield & Connell, 2022a), which casts doubt on the assumption that features are a good approximation of how people represent concepts. It would, therefore, be desirable to have an account of the basic‐level advantage in categorization that was not subject to the theoretically problematic assumptions of feature‐based representations.

### 1.1 Linguistic–sensorimotor theories of concepts and categorization

Recent research suggests that an alternative account of conceptual representation, based on both sensorimotor knowledge (i.e., perception, action, and affective experience of the world) and linguistic distributional knowledge (i.e., statistical patterns of words in language), offers a better explanation of the basic‐level advantage than accounts based on semantic features (van Hoef et al., 2023). Linguistic–sensorimotor theories hold that both linguistic distributional knowledge and sensorimotor simulation play mutually supporting roles in mental representations and learning (Barsalou, Santos, Simmons, & Wilson, 2008; Connell & Lynott, 2014; Davis & Yee, 2021; Louwerse, 2011, 2018; Vigliocco, Meteyard, Andrews, & Kousta, 2009), thus bridging the gap between symbolic (e.g., Fodor, 1975) and embodied (e.g., Barsalou, 1999; Zwaan, 2004) theories of cognition. All concepts, across both concrete and abstract domains, can be grounded in sensorimotor experience of perceptual modalities and/or action effectors (Banks & Connell, 2022b; Connell, Lynott, & Banks, 2018; Villani, Lugli, Liuzza, & Borghi, 2019), as can the referent concepts of all parts of speech to varying extents (Lynott, Connell, Brysbaert, Brand, & Carney, 2020; see also Connell & Lynott, 2012; Wingfield & Connell, 2022a). This sensorimotor grounding can be accumulated in a number of ways, including directly via perception and action or vicariously via witnessing other people's experiences or hearing stories (Barsalou, Niedenthal, Barbey, & Ruppert, 2003; Connell & Lynott, 2014). At the same time, linguistic distributional knowledge can accurately encode some—though not all—sensorimotor information involved in a concept (Connell, 2019; Louwerse & Connell, 2011; Louwerse & Jeuniaux, 2008; Riordan & Jones, 2011; see also Wingfield & Connell, 2022a) and can even help to ground concepts indirectly by bootstrapping meaning across words (Louwerse, 2011, 2018). Hence, concepts such as *bird* or *democracy* are represented—and may be accessed or activated—both through the distributional links of their concept labels with other words in language, and through partial replay of sensorimotor experiences associated with the concepts. Or in other words, both linguistic distributional and sensorimotor information provide distinct but partially overlapping sources of lexical semantics.

One of the key posited differences between the two forms of information is that linguistic representations (words, phrases, and their statistical associations) are computationally cheaper than sensorimotor representations (simulations of perception and action), being faster to activate and process (Barsalou et al., 2008; Connell & Lynott, 2014; Louwerse, 2011) and occupying fewer resources in working memory (Banks & Connell, 2024; Connell & Lynott, 2014; Dymarska, Connell, & Banks, 2022). In particular, the linguistic‐shortcut hypothesis (Connell, 2019; Connell & Lynott, 2014) specifies that these characteristics of linguistic‐distributional knowledge enable a heuristic mechanism whereby linguistic labels and their distributional patterns can, in some circumstances, stand in for a concept's detailed, grounded sensorimotor representation. Linguistic distributional information can be used to catalyze and steer conceptual processing, such as conceptual combination (Connell & Lynott, 2013; Lynott & Connell, 2010) and semantic feature generation (Santos, Chaigneau, Simmons, & Barsalou, 2011), with more computationally and resource‐demanding representations that involve sensorimotor simulations coming online only when necessary (Barsalou et al., 2008; Connell, 2019; Louwerse, 2018; see also Ferreira, Bailey, & Ferraro, 2002), modulated by the demands of the task (Connell, 2019; Connell & Lynott, 2014; Wingfield & Connell, 2022b).

Critically, the linguistic shortcut also applies to categorical cognition. In a category production task (i.e., freely generating members of a category, also known as *semantic fluency*), Banks, Wingfield, and Connell (2021) recently found that overlap in sensorimotor experience and linguistic distributional knowledge between a category concept (e.g., *dog*) and a given member concept (e.g., *Labrador*) independently predicted how early and often people produce that member concept for the category. Indeed, both linguistic distributional and sensorimotor contributed to category production to a similar extent, across both concrete and abstract categories. However, the relative importance of linguistic distributional information can vary according to the specific demands of the task (e.g., Connell, 2019). Category production is a verbal task where both the cue (written category name) and response (spoken category members) are words, meaning that it is perhaps unsurprising that linguistic distributional knowledge played such a major role. Category verification in a classic label → picture paradigm, however, combines a linguistic cue (written category name) with an image target (picture of object), which can reduce the role of linguistic distributional information relative to sensorimotor simulation (Louwerse & Jeuniaux, 2010). Indeed, in such a category verification task (e.g., label *dog* → [image of a Labrador]), van Hoef et al. (2023) found that overlap in sensorimotor experience was a strong predictor of the timecourse of categorization (i.e., participants were quicker to verify objects that strongly overlapped in sensorimotor experience with their category concept) and the categorization decision itself (i.e., yes/no judgment of category members), whereas overlap in linguistic distributional information had an additional, albeit smaller, independent effect. Importantly, these linguistic–sensorimotor predictors overall *outperformed* discrete taxonomic levels (i.e., superordinate, basic, superordinate) in explaining the basic‐level advantage in category verification. That is, rather than assuming that basic‐level categories are privileged as the entry point to semantic memory (Jolicoeur et al., 1984) or as the degree of abstraction that optimally differentiates members from nonmembers via feature similarity (Murphy & Brownell, 1985), van Hoef and colleagues showed that performance can be more precisely predicted by the degree of overlap in sensorimotor experience and/or linguistic distributional knowledge between category and member concepts. The basic‐level advantage may thus emerge as an overall behavioral artifact of this linguistic–sensorimotor overlap, but only at the global level (i.e., across a set of items) rather than for every object concept individually.

Van Hoef et al. (2023) describe a potential linguistic–sensorimotor process model of the basic‐level advantage in category verification, which shares many similarities with the preparation model proposed by Murphy and Smith (1982) but substitutes their discrete functional and perceptual features for sensorimotor and linguistic distributional knowledge. Like the original preparation model, the sensorimotor account does not assume any taxonomic level is privileged, but rather that performance is exclusively driven by the degree of overlap in sensorimotor experience and/or linguistic distributional knowledge between category and member concepts. According to the linguistic–sensorimotor preparation model, therefore, when participants see a category label (e.g., *dog*), it activates a sensorimotor simulation of the referent concept as well as linguistic distributional knowledge about its contextual neighbors in language. When the participant then sees the subsequent image (e.g., a *Labrador*), it too activates a sensorimotor simulation of the depicted object as well as the object name itself (i.e., a linguistic label) and accompanying linguistic distributional information. The participant then attempts to verify whether the representation activated by the pictured object matches the representation activated by the category label and will respond “yes” if the sensorimotor and/or linguistic information achieves a close enough match. The greater the sensorimotor overlap between category and object concepts, the more useful the preactivated sensorimotor simulation of the category label is to simulating the depicted object (i.e., the less additional activation is required) and the faster and more accurate the response. Similarly, the greater the overlap in linguistic distributional knowledge between category and object labels, the greater the probability that the category label will have preactivated the object label as a distributional neighbor, and the faster and more accurate the response. However, although the linguistic–sensorimotor preparation model accounts for van Hoef et al.’s empirical results, it has thus far remained a verbal theory and has not yet been formally tested in a computational model.

### 1.2 The current study

Theories of process and representation in cognitive psychology—including those on object categorization—are frequently first formulated verbally (i.e., informally). Defining theories informally has distinct benefits, as it allows researchers to communicate and reason about ideas at a higher level without needing first to fully pin down every precise detail and ramification. However, to work *exclusively* with informal theories also has clear drawbacks. In the absence of a formal specification whose predictive consequences can be made precise, we risk theories which are not falsifiable, contain hidden assumptions, or are even internally inconsistent (Guest & Martin, 2021; Borsboom, van der Maas, Dalege, Kievit, & Haig, 2021; see also Cooper & Guest, 2014). Thus, in the pursuit of robust, testable theory‐building, it is ultimately insufficient to deal only with verbal definitions. Such formalized testing has been attempted for broader theories of categorization (e.g., Minda & Smith, 2001; Nosofsky, 1987; Nosofsky & Palmeri, 1997; Pothos & Chater, 2002; Smith & Minda, 2000). With regard to the basic‐level advantage in categorization, a handful of attempts have been made at modeling its emergence during concept formation (e.g., Rogers & McClelland, 2004; Quinn & Johnson, 1997; 2000). While these models have provided valuable ideas about how some concepts and categories might develop in infants under different assumptions, their interpretations and psychological validity have been questioned (Murphy, 2016), and they do not necessarily scale up to categorical processing in adults. A formal computational test has been done on the role of linguistic‐distributional and sensorimotor knowledge in category *production*, where Banks et al. (2021) went beyond a verbal theory and implemented their theoretical framework in a computational model. Results of model simulations confirmed the importance of spreading activation in both linguistic distributional and sensorimotor knowledge, where the model output of activated member concepts was indistinguishable from a typical human. However, the process of category verification is different from that of category production, and the linguistic–sensorimotor explanation of the basic‐level advantage requires computational instantiation and testing in its own right.

It is in this spirit that we proceed in the present paper. We specify a computational model which implements a linguistic–sensorimotor account of the category‐verification task (i.e., van Hoef et al., 2023, linguistic–sensorimotor preparation model), building on a previous computational model we developed for category production (Banks et al., 2021). In a series of simulations across multiple datasets, we demonstrate that our computational model performs the task of category verification to a level comparable with human participants, and furthermore that its operation naturally gives rise to the basic‐level‐advantage phenomenon. In so doing, we corroborate the findings of van Hoef et al. (2023) that linguistic‐distributional and sensorimotor information both contribute to categorical cognition, and demonstrate that the basic‐level advantage can at least in part emerge naturally from the structure of language and sensorimotor experience.

## 2 Model specification

We developed a computational model of category verification which makes concrete the notion that the conceptual system comprises an interactive combination of linguistic distributional and sensorimotor knowledge. The model is intended to cover a full‐size conceptual system which approaches that of an adult, educated native speaker of English (i.e., approximately 40,000 concepts: Lynott et al., 2020; Brysbaert, Warriner, & Kuperman, 2014). In principle, it captures a snapshot of the conceptual system at the point of commencing an individual category verification trial; that is, we do not attempt to model how the nature of conceptual representations changes dynamically with time and experience, but that does not mean we assume conceptual knowledge is static (Connell & Lynott, 2014). The computational model we present here is an evolution of the one first described in Banks et al. (2021), where it was employed in modeling a category production task (see also Banks & Connell, 2022a) and successfully captured both the likelihood and ordinal rank of producing a given category member. The present form of the model implements the spreading activation and decision processes involved in a label → picture category verification task of the kind often used to elicit the basic‐level advantage in categorization (e.g., Rosch et al., 1976; Tanaka & Taylor, 1991) and specifically implements the assumptions of the linguistic–sensorimotor preparation model (van Hoef et al., 2023).

**Fig. 1:**
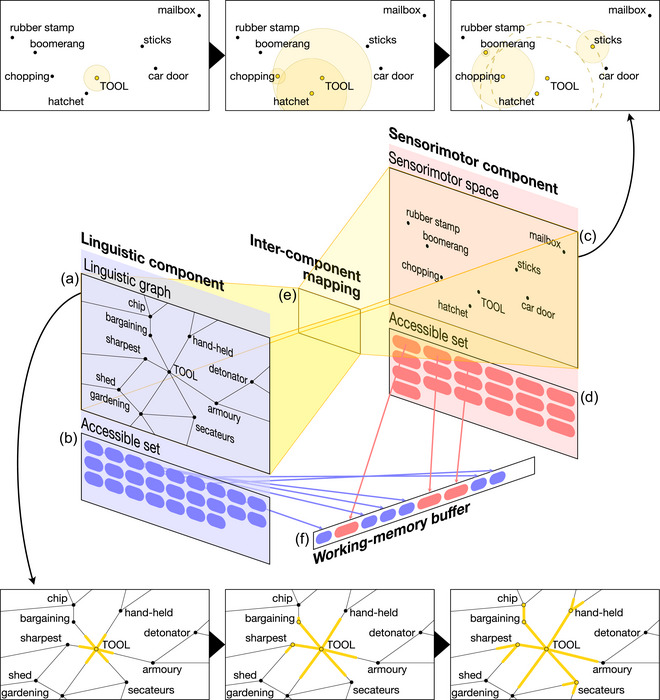
Schematic representation of the computational model; items and distances are illustrative only. The linguistic component of the model comprises the linguistic graph (A) across which activation spreads (bottom panels), together with a set of currently activated items (B); as the set fills, it attenuates further activation within the graph. The sensorimotor component of the model comprises the sensorimotor space (C) through which activation spreads (top panels), together with a set of currently activated items (D); as this set fills, it attenuates further activation within the space. The linguistic and sensorimotor components interact via item‐level mappings (E). Items sufficiently activated in either component enter a shared working‐memory buffer (F).

In brief, the model comprises a dual‐component structure: one component for linguistic distributional knowledge that is implemented as a graph (i.e., word nodes connected by edges), and another component for sensorimotor knowledge that is implemented as a continuous, multidimensional space (derived from the Lancaster Sensorimotor Norms: Lynott et al., 2020). A schematic depiction of the model structure is shown in Fig. 1. Critically (and advancing the architecture of other versions: Banks et al., 2021), the linguistic and sensorimotor components interact by means of a bidirectional mapping at the concept level (e.g., the *dog* node in the linguistic graph maps to the *dog* concept in sensorimotor space) as per linguistic–sensorimotor theories of the conceptual system (Barsalou et al., 2008; Connell & Lynott, 2014; Louwerse, 2011). As a result, all representations in the model are fully grounded, and activation can spread between neighboring concepts (e.g., from node to node in the linguistic component, or across multidimensional space in the sensorimotor component) *and* between word labels and their referents (e.g., from the linguistic *tool* node to the sensorimotor concept *tool*, and vice versa). By activating a category label (e.g., linguistic node *dog*) followed by a target concept (e.g., *Labrador*), the model can verify category membership by checking if the target activation level clears a given threshold.

In the following sections, we outline the model framework and describe the implementation of the category verification task, before presenting simulation experiments that examine the presence of the basic‐level advantage in model performance and compare it to human datasets. Model code and parameter details are available on OSF at https://osf.io/m5hjb.

### 2.1 Linguistic component

The linguistic component includes all parts of the model which describe the spreading of activation between concepts through linguistic distributional connections. The primary part of this component is a graph of linguistic nodes (words) that are connected by edges to their distributional neighbors (Fig. 1A), an identical construction to that used by Banks et al. (2021). Linguistic‐distributional information was operationalized using a corpus of 200 million words of British‐English television and film subtitles (see van Heuven, Mandera, Keuleers, & Brysbaert, 2014). There are 40,000 nodes in the graph, labeled by the 40,000 most frequent tokens in the corpus. Edges connect nodes according to their first‐order co‐occurrences within a 6‐gram window across the corpus, using positive pointwise mutual information (PPMI: Bullinaria & Levy, 2007),^2^ so that only words which co‐occur at a rate higher than chance (i.e., nonzero PPMI) are connected by an edge. Hence, the graph contains 26,334,191 edges rather than the 800 million edges that a fully connected graph would require. Edge length is inversely related to the PPMI 6‐gram score, so that the pair of words with the lowest nonzero PPMI score is given the longest edge, and the pair of words with the highest PPMI score is given the shortest edge, with intermediate edge lengths given by a negative linear relationship between the extremes. That is, the higher the rate of co‐occurrence between two words, the shorter their corresponding edge length.

Activation is allowed to spread along the edges of this graph using the following algorithm. Incoming activation can accumulate in a word node. Words which become activated above a fixed *firing threshold* propagate a copy of their activation outward along all connected edges, traveling a fixed distance with each iteration of an internal clock. Activations of nodes, and activations traveling along edges, decay over time according to exponential and Gaussian decay functions, respectively (cf. Schweickert & Boruff, 1986). Words which are activated above the firing threshold also enter a *linguistic accessible set* (Fig. 1B), representing the set of concept labels retaining a memory trace in the context of an ongoing task. New activations within the linguistic component are attenuated linearly by the number of items currently in the accessible set, such that no new activations could occur when the set reached a *maximum capacity* of 3000 items.^3^ Activation levels of individual nodes are capped at 1.0.

### 2.2 Sensorimotor component

The sensorimotor component includes all parts of the model which describe spreading activation between concepts through a multidimensional space of sensorimotor experience. The primary part of this component is metric space (Fig. 1C) defined by sensorimotor distance (Wingfield & Connell, 2022a) between the 39,707 named concepts in the Lancaster Sensorimotor Norms (LSN; Lynott et al., 2020). This space is identical to the one used in the model of Banks et al. (2021). The LSN provides a vector per concept of sensorimotor strength ratings across 11 dimensions total: five dimensions of action experience (using the hand/arm, foot/leg, torso, mouth/throat, and head excluding mouth/throat) and six dimensions of sensory experience (visual, auditory, haptic, olfactory, gustatory, interoceptive). We use the Minkowski‐3 distance^4^ between each pair of these concepts (see Wingfield & Connell, 2022a) to compare points in the space, so that concepts which have very similar vectors of sensorimotor strength ratings in the LSN are located close together in the space, and those with very dissimilar vectors are located far apart.

Activation is allowed to spread throughout the space according to the following algorithm. Incoming activation can accumulate in a concept point. Concepts which became activated above a fixed *propagation threshold* send a copy of their activation to radiate uniformly throughout the space, expanding in a multidimensional “sphere” whose radius increases by a fixed Minkowski‐3 distance in each iteration of the model clock. Any other concept points which are met by this expanding sphere become activated themselves by the amount of activation which reaches them. Concept points which become activated above the propagation threshold enter a *sensorimotor accessible set* (Fig. 1D), representing those concepts whose sensorimotor representations retain a memory trace in the context of the present task. When items first enter the accessible set, they radiate new spheres of activation in the space. Spheres of activation which achieve a set *maximum radius* are removed, so that activation of one concept can directly activate only its close neighbors in the space. Activation of concept points decay according to a log‐normal decay function over time (see Mueller & Krawitz, 2009). New activations within the component are attenuated linearly by the prevalence of the concept label (i.e., how well‐known the word is among native speakers; Brysbaert, Mandera, McCormick, & Keuleers, 2019) in order to approximate how well‐practiced people might be at simulating the referent concept (i.e., seldom‐simulated concepts receive weaker activation than concepts that are simulated often: see Banks et al., 2021). New activations are also attenuated linearly by the number of concepts currently in the accessible set such that no new activations can occur when the set reaches a set *maximum capacity* of 3000. Activation levels of individual concepts are capped at 1.0.

### 2.3 Intercomponent mapping

When a concept in one component is sufficiently activated (i.e., above a fixed cross‐component propagation threshold: see Supplementary Materials for parameter settings), activation is allowed to spread after a short delay^5^ to the corresponding concept(s) in the other component according to a fixed mapping (Fig. 1E) between linguistic graph nodes (i.e., word labels) and sensorimotor concept points (i.e., concepts with those labels in the LSN). However, mappings are complicated by dialectal differences between components because the labels of sensorimotor concepts in the LSN (as given in Lynott et al., 2020) were primarily American English, whereas the linguistic component was built from a corpus of British English. In order that the model could operate consistently within a single dialect, which we selected to be British English to match the dialect of the target human population (see Simulations), we translated where possible the labels of sensorimotor concept points into their British‐English equivalents. Examples of such translations included spelling variants (e.g., *anesthetic* → *anaesthetic*, *conceptualize* → *conceptualise*) and vocabulary differences (e.g., *sidewalk* → *pavement, ladybug* → *ladybird*); full details are given in Supplementary Materials on OSF (Table S2). Following this translation process, both components of the model could operate in a common dialect of British English; we hereafter report examples of sensorimotor concepts using British‐English spellings.

Intercomponent mappings are often, but not absolutely, one‐to‐one. Words in the linguistic component are mapped directly to their referent sensorimotor concept points where such concept points exist (e.g., linguistic word *cat* is mapped onto the sensorimotor concept *cat*). Where a word does not have an identically labeled sensorimotor concept, it is often because the sensorimotor concept exists only in uninflected forms (e.g., *cat* is a sensorimotor concept label, but *cats* is not). Therefore, where the word is an inflected noun or verb, we attempt to map to the uninflected lemma in the sensorimotor component (e.g., *cats* → *cat*, *walked* → *walk, rivalries* → *rivalry*) using the WordNet lemmatizer included in the Python Natural‐Language Toolkit (NLTK; Bird, Klein, & Loper, 2009). Concepts in the sensorimotor component are mapped directly onto words in the linguistic component where those words exist. Where the word does not exist in the linguistic component, it is often because the sensorimotor concept label is a multi‐word phrase (e.g., *quality control*), in which case the sensorimotor concept is mapped to each of its constituent word nodes in the linguistic component (e.g., *quality* and *control*); this form of mapping occurred for 2673 (6.7%) sensorimotor concept points. Finally, where multiple spelling variants exist in the linguistic component, the sensorimotor concept is mapped to all such nodes (e.g., the sensorimotor concept point *yoghurt* is mapped to both linguistic nodes *yoghurt* and *yogurt*). In any cases where a concept from one component is mapped to multiple concepts in the other, spreading activation is split evenly between the target concepts. In this way, 63.7% of linguistic nodes are mapped to corresponding sensorimotor concept points (i.e., their referent concepts have full sensorimotor vectors in the LSN), and 51.8% of sensorimotor concepts have a mapping to a linguistic node (i.e., their labels are among the 40,000 most frequent words in the corpus). Any concepts that lack intercomponent mappings (e.g., sensorimotor *precognition* has no linguistic counterpart; linguistic *unionist* has no sensorimotor counterpart) can still be activated via their intracomponent neighbors, meaning that all words in the linguistic component can be grounded either directly or indirectly (Louwerse, 2018; see also Connell, 2019). That is, the model implements a full‐size, fully grounded, adult conceptual system.

### 2.4 Working memory buffer

Concepts that become activated in either component compete for entry into a *working memory buffer* (Fig. 1F)—a list of the most‐activated concepts in both components—which theoretically represents the limited store of items that are currently available to conscious awareness (similar to Baddeley's, 2000, episodic buffer). To enter the buffer, a concept's activation must exceed the *buffer threshold*, where it displaces concepts already in the buffer if they have a lower activation level. Ties were resolved according to recency of entry to buffer, followed by recency of activation emission from the concept point or node, followed by the original load order of concepts into the model (alphabetical; an implementational convenience). As a result, the buffer effectively operates a first‐in‐first‐out replacement mechanism. The capacity of the buffer is fixed, but—following findings that linguistic representations occupy less working memory capacity than their sensorimotor referents (Dymarska et al., 2022; see also Banks & Connell, 2024; Connell & Lynott, 2014)—linguistic concepts occupy less “space” in the buffer than sensorimotor concepts. When full, the buffer can hold a maximum of nine sensorimotor items or 12 linguistic items (based on capacity estimates for familiar object concepts: Dymarska et al., 2022). When a concept's activation decays below the threshold, it is removed from the buffer.

### 2.5 Performing the category verification task

To perform the label → picture category verification task using the above framework, we aimed to model the typical experimental trial structure. A trial starts with the *cue stage*: an initial presentation of a word labeling a category (e.g., “dog”) that lasts for a particular period of time (the stimulus‐offset asynchrony: SOA). Next comes the *target stage*: the presentation of an image depicting either a category member (e.g., a Labrador) or a nonmember (e.g., a teacup). Upon image presentation, the task of the participant (or model) is to respond “yes” if the depicted object is a member of the named category, or “no” if not.

**Fig. 2:**
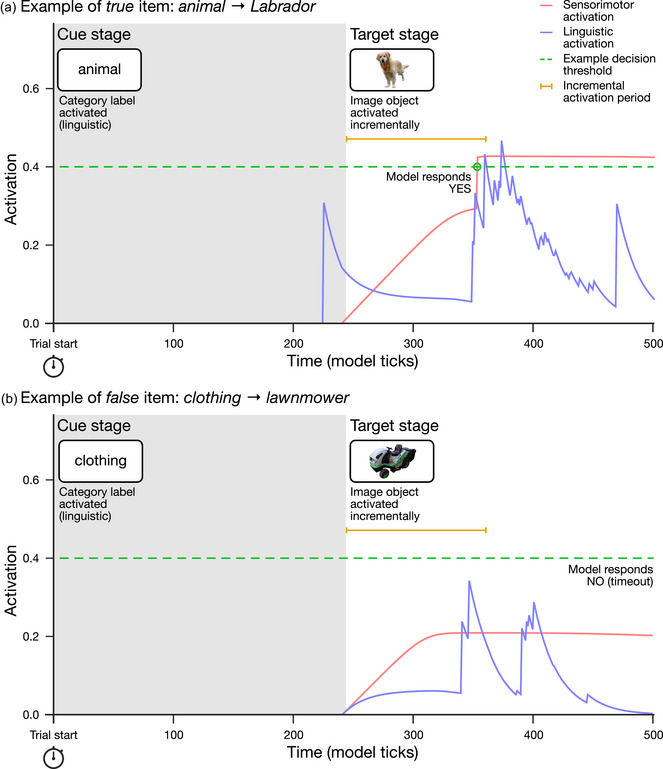
Examples of target object activations over time in the sensorimotor (red line) and linguistic (blue line) components during the modeling of an individual trial. The dotted green line shows a sample decision threshold: if activation in either component crosses the threshold, then a “yes” decision is made (Panel A), otherwise a “no” decision results (Panel B).

Therefore, for each trial (i.e., each label → picture item), we first set all activations in the model to zero, emptied the accessible sets and buffer, and reset the model clock, so that the state of the model was identical at the start of each trial. Model operation then proceeds as follows (see Fig. 2). We begin the cue stage by seeding an activation value of 1.0 (the maximum) at the node representing the category name in the linguistic component (e.g., *animal*), since the category label cue is presented lexically. We then iterate the model clock, allowing activation to propagate freely throughout and between both components for a given number of ticks that represents the SOA (see Simulation 1 for details of SOA setting). At the end of the SOA, the target stage begins,^6^ where we apply an incremental activation to the object concept depicted in the target picture (e.g., *Labrador*) in the sensorimotor component (i.e., to represent gradual recognition of the depicted object by retrieving it from long‐term memory) *and* in the linguistic component (to allow for the early, automatic activation of object names during recognition: e.g., Mani & Plunkett, 2010; Meyer, Belke, Telling, & Humphreys, 2007).

Critically, from the start of the target stage, we monitor the activation level of the object concepts in both components to determine the critical trial decision (i.e., category verification yes/no). If the target object activation in either component exceeds a given decision threshold, the model produces a response of *yes* (i.e., decides that the object in the image *was* a member of the named category). If the target object activation fails to exceed the threshold by the end of the target stage (i.e., when the model clock reaches 500 ticks, a long enough duration to allow all target activations to peak and decay), the model produces a response of *no* (i.e., decides that the object in the image *was not* a member of the named category).

The entire process of cue and target stages is repeated afresh for each item, with the model retaining no “memory” or state from previous trials. In essence, the closer the category and object concepts in either the linguistic graph or sensorimotor space, the stronger the trace activation that propagates from the category to the target object, and the more likely it is that target object activation will exceed the decision threshold. Where spreading activation from the category concept can reach a nearby object concept, the activation level of the object concept is boosted and a “yes” decision becomes more likely (see Fig. 2 *animal → Labrador*). On the other hand, where spreading activation from the category concept dissipates before reaching a distant object concept, the activation level of the object concept is limited to that applied at the target stage, and a “no” decision is likely (see Fig. 2 *clothing → lawnmower*). Such yes/no decisions from the model, examined across the full range of decision thresholds (0–1), allow us to compare its performance directly with that of human participants in the various datasets we model in Simulations 1 and 2.

## 3 Simulation 1: Category verification in a yes/no decision paradigm

The goals of this first simulation were to examine the model's ability to perform category verification, including whether it categorized at the basic level more accurately than at the superordinate level, and to compare its performance to human datasets of label → picture category verification that produced the basic‐level advantage (taken from van Hoef et al., 2023). In this way, we aimed to test the linguistic–sensorimotor preparation model of the basic‐level advantage.

We hypothesized that the model would successfully perform category verification, accepting category members and rejecting category nonmembers at a level above chance, and that it would categorize at the basic level more accurately than at the superordinate level (i.e., the most robust behavioral manifestation of the basic‐level advantage). We also hypothesized that the probability of an individual object being verified as a category member by human participants would relate to the peak activation reached by that object's representation in the model. The sensorimotor–linguistic preparation model (van Hoef et al., 2023) states that the greater the overlap between category and object concepts in sensorimotor experience and/or linguistic distributional knowledge, the more useful the preactivated sensorimotor simulation of the category label will be to simulating the pictured object and the more accurately the object will be categorized. As a result, the closer an object concept is to a category concept in linguistic and/or sensorimotor terms (e.g., the closer *Labrador* is to *animal*), the more likely humans are to verify it as a category member (i.e., higher proportion of “yes” responses) *and* the greater the activation it will receive from the category concept during the model's cue stage (i.e., higher peak activation level).

All data and analyses are available on OSF at https://osf.io/m5hjb.

### 3.1 Method

#### 3.1.1 Materials

Items consisted of 173 category → object pairs, which were all extracted from Experiments 1 and 2 in van Hoef et al. (2023). Of these items, 115 were true and 58 were false.^7^ True items consisted of a basic or superordinate category label paired with an actual member concept (e.g., basic *dog → Labrador*; superordinate *animal → Labrador*). False items comprised a basic or superordinate category label paired with a nonmember concept (e.g., basic *snake → elephant*; superordinate *clothing → lawnmower*) and acted as filler items in the original van Hoef et al. study. To maximize item availability in the model and avoid excluding items unnecessarily, we replaced low‐frequency spellings (e.g., *mocassin* changed to *moccasin*) and substituted punctuation or dialectic equivalents (e.g., *rollerskate* changed to *roller‐skate*). We opted to focus on basic‐ and superordinate‐level categories only, and not include subordinate‐level categories, for two reasons. First was because the basic versus subordinate advantage does not always emerge robustly in category verification (e.g., Rosch et al., 1976; see also van Hoef et al., 2023, Experiment 2). Second, because target presentation involved activating the object name/concept rather than a visual image (e.g., subordinate items seen by participants such as *Labrador* → [picture of Labrador] would be presented to the model as *Labrador → Labrador*), subordinate category labels would directly preactivate the object label and, therefore, artificially boost model accuracy. For separate analysis per categorical level, there were 82 basic‐level items (51 true, 31 false) and 91 superordinate‐level items (64 true, 27 false).

Human category verification data for these items comes from two behavioral experiments: Dataset 1 from a task run in a traditional lab setting (van Hoef et al.’s Experiment 1a) and Dataset 2 from a direct replication (i.e., using the same stimuli) run online via a web‐based experimental platform (van Hoef et al.’s Experiment 2a). Both experiments followed the classic paradigm described in Rosch et al. (1976), where participants are presented with a category label followed by a picture and are asked to judge whether the pictured object belongs to the category described by the preceding label by pressing a key for either “yes” or “no” (i.e., a 2‐alternative forced‐choice, 2AFC, paradigm). For comparison against the model, we calculated for each participant the proportion of “yes” responses given to both true and false items, representing that participant's hit rate and false‐alarm rate, respectively; see Supplementary Materials on OSF. For each category → object item, we also calculated the mean proportion of “yes” responses given by participants in each dataset, representing the likelihood of human participants accepting that item as a category member.

#### 3.1.2 Computational model

Four category labels and 20 object labels comprised multi‐word terms (e.g., *letter opener*); in modeling these trials, we divided activation equally between all constituent concepts (i.e., *letter* and *opener*), and, in the case of multi‐word object labels, based the model decision on activation level of either constituent concept (e.g., a “yes” decision occurred if activation of either *letter* or *opener* exceeded the decision threshold). For each item, we recorded the final model decision (i.e., “yes” or “no”) and the peak activation level achieved by the target object concept.

We set the SOA duration at 241 model ticks, which is sufficient time for activation to traverse the longest straight‐line distance from a category to its target object in both linguistic and sensorimotor components. That is, every target object in the present item set could in theory receive some preactivation from the category during the cue stage, prior to being directly activated in the target stage.^8^ We iteratively adjusted some model parameters (e.g., firing and entry thresholds, node decay rate, cross‐component propagation delays) to maximize the model's performance relative to human Dataset 1 (see Supplementary Materials, Table S1, for the list of parameters we optimized during Simulation 1). Parameters were then locked and no further adjustments were made in subsequent simulations; we validate the model against a new set of items in Simulation 2.

#### 3.1.3 Evaluating model performance

We used receiver operating characteristic (ROC: Metz, 1978) curves to visualize and analyze binary category verification decisions by plotting the hit rate (i.e., proportion of true items with “yes” responses) against the false‐alarm rate (i.e., proportion of false items with “yes” responses) across the range of possible decision thresholds. For a random classifier (i.e., chance performance), the hit rate would tend to equal the false‐alarm rate, and thus the ROC curve resembles the identity line. The area under the curve (AUC) of an ROC curve provides a summary measure of the ability of a classifier to select hits without admitting false alarms (Hanley & McNeil, 1982), and is often a more informative statistic than simple accuracy, especially when there are unequal numbers of items within classes (Fawcett, 2006). A perfect classifier would hence have an AUC of 1.0, a random classifier would tend to have an AUC of 0.5, and a perfect anticlassifier would have an AUC of 0.0. By calculating ROC‐AUC for the model *and* for each individual participant (see, e.g., Jiang et al., 2019), we were able to compare the performance of the model at a full range of decision‐threshold values to the performance of human participants who varied in their categorization abilities.

To assess whether the model behavior exhibited the basic‐level advantage, we split the data by categorical level into basic‐ and superordinate‐level items, reran the ROC‐AUC analysis on each subset, and compared their performance (i.e., whether model ROC‐AUC is better for basic‐level than superordinate‐level items).

Finally, for each item, we compared the peak activation attained by the target object concept in the model to the likelihood of participants responding “yes” (i.e., hit rate for a true item, false alarm rate for a false item). Specifically, we correlated model peak activation with human “yes” likelihood using a one‐sided alternative hypothesis of a positive correlation for which we report the log Bayes Factor (JASP v0.16.3: JASP Team, 2022). We also evaluated the strength of the correlation using bootstrapped confidence intervals (computed in Python using the Seaborn package v0.11.1: Waskom, 2021).

### 3.2 Results

**Fig. 3:**
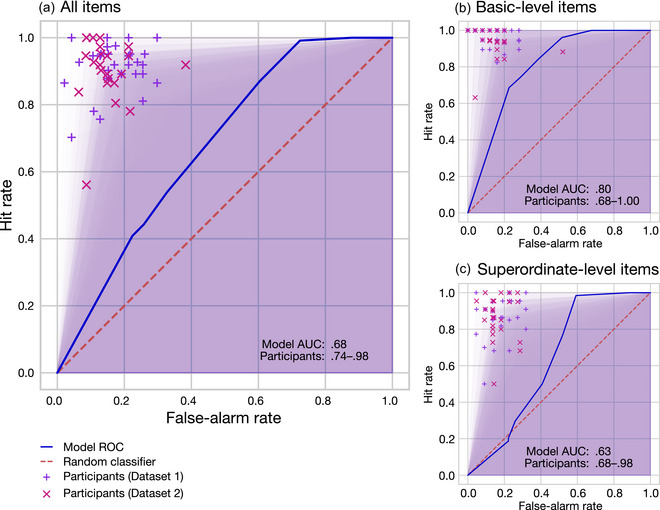
ROC curves of Simulation 1 category verification performance of model versus human participants for all items (Panel A), basic‐level items (Panel B), and superordinate‐level items (Panel C). Model ROC curve (solid blue line) is plotted across decision thresholds 0.0–1.0 compared to chance performance of a random classifier (dotted red line). Performance of each individual participant is indicated by a cross, with the area under the curve (AUC) shown as a shaded background region. Inset numbers per panel report AUC for the model and the range of AUC values for human participants.

Using the ROC‐AUC measure, the model successfully performed category verification, with all‐item AUC = .68. This level of performance was below the participant AUC range [.74, .98], indicating that—while humans varied greatly in their categorization abilities—they were overall better than our model at verifying category membership of these items. The all‐item ROC curves for model and participants are shown in Fig. 3A.

Nonetheless, when the item set was split by taxonomic level of the category label, the model (as we predicted) exhibited an emergent basic‐level‐advantage, where model AUC = .80 for basic‐level items was markedly better than AUC = .63 for superordinate‐level items. Indeed, the magnitude of the basic‐level advantage was larger for the model (mean AUC difference = .17) than for participants (mean AUC difference = .05). In the case of the basic‐level items, the model AUC of .80 sat comfortably within the participant range [.68, 1.0]; that is, the model could categorize at the basic level about as well as people could (see Fig. 3B). In the case of superordinate‐level items, however, model AUC of .63 was below the participant range [.68, .98], meaning that the model found superordinate‐level categorization more difficult than did people (see Fig. 3C).

**Fig. 4:**
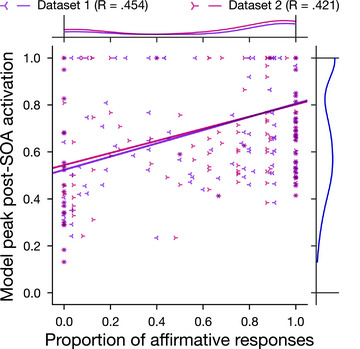
Scatterplot of peak activation level attained by each object concept within the model against the proportion of human participants who responded “yes” (i.e., accepted that object as a category member) in each Simulation 1 dataset. Marginal plots depict distribution densities. *R*‐values relate to linear trendlines per dataset.

Finally, as expected, the peak activation level reached by target object concepts in the model was correlated with the likelihood of participants responding “yes” (for Dataset 1: *r* = .45, log BF_10_ =  17.86; for Dataset 2: *r* = .42, log BF_10_ =  14.82). That is, the more strongly the category label boosted activation of an object concept in the model (e.g., *flower* → *tulip*), the more likely people were to accept that object as a category member (Fig. 4).

### 3.3 Discussion

The model successfully simulated the category verification task, achieving a level of performance which was well above a chance classifier. Critically, the model's behavior exhibited the basic‐level advantage: objects preceded by a basic‐level category were easier for the model to correctly verify (higher hit‐rates in comparison to false‐alarm rates) than those preceded by a superordinate‐level category label. This phenomenon emerged in the model's behavior despite the fact that the model itself is not equipped with any knowledge of taxonomic hierarchies. In performing the category‐verification task, it relied solely on how accessible the objects shown in the image were from the category label via spreading activation across linguistic‐distributional and sensorimotor components. Results of this simulation, therefore, support van Hoef et al. (2023) linguistic–sensorimotor preparation model of the basic‐level advantage. Specifically, the model findings corroborate the idea that both linguistic and sensorimotor knowledge are relevant to the category‐verification task, and that the relationship between category and member concepts in linguistic and/or sensorimotor terms is key to explaining the effect of the basic‐level advantage effect.

Nonetheless, the model's performance at category verification was overall not as good as that of human participants. Participants were in general very good at the task, with high hit‐rates and reasonably low false‐alarm rates across items as a whole (see Fig. 3; full statistics can be found in Table S3). However, when we broke items down by the taxonomic level of categorization required (i.e., basic level or more generalized superordinate level), we found that the participants’ performance advantage was largely concentrated on superordinate‐level items: when restricted only to basic‐level items, the model achieved a performance which was within the range of the participant group. That is, regardless of whether an object was a true member of a category, the peak activation of the object concept within the model substantially predicted the likelihood that participants accepted that object as a category member (i.e., responded in the affirmative). Not only did the model generally replicate the successes of human participants (e.g., both model and people tended to accept *salmon* as a member of the category *fish*), but it also made many of the same mistakes (e.g., both model and people tended to accept *bottlenose* [dolphin] as a member of the category *fish*).

In this simulation, the values for the model's free parameters were adjusted to optimize its performance on category verification items taken from van Hoef et al. (2023). The risk exists that the model's parameters may in some sense have been overfit to the particular items used in the simulation,^9^ and that the observed effects would not generalize to category verification of novel items. Moreover, the model's operation uses a single threshold as the basis of the category verification decision (i.e., responding “yes” if object activation cleared the threshold but “no” otherwise), which is closer to a go/no‐go verification paradigm than the 2AFC (i.e., yes/no) paradigm used by van Hoef and colleagues. For these reasons, we conducted an additional simulation to verify that the performance of the model would generalize, based on a fresh dataset of human performance using a go/no‐go version of the label → picture category verification task.

## 4 Simulation 2: Category verification in a go/no‐go paradigm

To rule out the possibility that we had overfit the model's parameters to the particular item set used in Simulation 1, we conducted a validation study using a new item set and a modified category verification paradigm. The decision procedure implemented in the model makes an affirmative “yes” response when the decision threshold is exceeded, or an implicit “no” if it was not exceeded by the end of the trial. To match this decision procedure more closely, we designed and collected data for a label → picture category verification study using a go/no‐go paradigm (i.e., participants should respond “yes” if the pictured object is a member of the category, or do nothing if it is not). In this way, both participants and model would in effect be making the same decision (to answer in the affirmative or make no affirmative response). We expected the go/no‐go task to elicit the basic‐level advantage (i.e., people will categorize more accurately at the basic level than at the superordinate level); our model predictions remained the same as in Simulation 1.

All materials, data, and analyses are available on OSF at https://osf.io/m5hjb.

### 4.1 Methods

#### 4.1.1 Human experiment

##### 4.1.1.1 Participants

For this preregistered study (https://aspredicted.org/2bpr‐kfcv.pdf), 30 participants (20 female; *M*_age_ =  36.4, *SD* =  11.5) took part via Prolific.co, an online crowdsourcing platform for the sum of £4.50 (approximately £9.00/h pro rata for an assumed duration of 30 min). On average, participants took 19 min to complete the task. Through Prolific's recruitment filter settings, we ensured that all participants were native speakers of English and had no dyslexia. Participants provided informed consent, which included consent to share all anonymous data in a public repository, and an acknowledgment that payment was contingent on passing data quality checks (i.e., participants were required to give a “go” response to at least two items in each block, and to pass 10 out of 12 of arithmetic attention checks). We replaced six participants for not meeting these preregistered criteria. An additional two participants were replaced due to technical malfunctions.

Sample size was determined through sequential hypothesis testing using Bayes Factors (Schönbrodt, Wagenmakers, Zehetleitner, & Perugini, 2017), where evidence for/against a hypothesis may accumulate until a predetermined evidence threshold is reached. We stopped sampling at N_min_ =  30, when our analysis indicated evidence for a basic‐level advantage in the accuracy data had cleared the specified threshold of BF_10_ ≥ 5 (actual BF_10_ =  3.69×10^37^) for two consecutive participants (i.e., balanced across lists).

##### 4.1.1.2 Materials

Test items consisted of 80 objects which were paired in full rotation with 12 category labels (8 basic‐level: *bird, boat, box, car, cup, dog, flower, tree*; 4 superordinate‐level categories: *animal*, *container*, *plant*, *tree*), to form 960 category → object items (160 true, 800 false). We grouped these items into category blocks, and divided each block evenly across two lists so that each list contained the same 12 category blocks with a different set of 40 items. Within each list, each basic‐level block contained 35 false items (e.g., *dog* → *goblet*) and 5 true items (e.g., *dog* → *Labrador*), while each superordinate‐level block contained 30 false items (e.g., animal → gondola) and 10 true items (e.g., animal → robin).^10^ For each target object, we retrieved two images with the lowest naming uncertainty (h‐statistic) in the van Hoef, Lynott, and Connell (2024) picture‐naming norms, and randomly paired each image with an occurrence of the object in different category blocks.

##### 4.1.1.3 Procedure

We ran the experiment on the online platform Gorilla.sc (Anwyl‐Irvine, Massonnié, Flitton, Kirkham, & Evershed, 2020), which handled both collection of informed consent and experimental data collection. Participants were randomly assigned to one of the two stimulus lists. Instructions told participants they would see a number of blocks which would start with a word followed by a series of object images, where the word represented a category and the images potential members of that category. Participants were asked to judge, for each image, whether it displayed a member of the category described by the word at the beginning of the block, and to press the spacebar as quickly as they could *only* when the image showed a category member. Trials were presented on a white background, in 12 blocks of 40 trials each. Each block started with a fixation cross for 200 ms, followed by the presentation of the category label (centered, black lowercase Arial, 64 points) for 1000 ms, followed by all 40 trials in random order. Each trial began with a blank screen displayed for 500 ms, followed by a fixation cross for 200 ms, and finally, the object image, which remained on screen for 1000 ms or until the participant pressed the spacebar. We measured response accuracy for both true and false items. After each block, participants were allowed a 15‐s break before the next block commenced.

##### 4.1.1.4 Analyses

We determined whether a basic‐level advantage could be distinguished in the data by running a mixed effects logistic regression of accuracy (miss/false alarm =  0, hit/correct rejection =  1) with crossed random effects of participant and nested object/image and a fixed effect of taxonomic level of the category label (0 =  basic, 1 =  superordinate). We used Bayesian model comparisons (Bayes Factors calculated from BIC; Wagenmakers, 2007) to test whether the data favored a model containing our fixed effect of taxonomic level over a model containing only random effects. We inspected coefficients to determine the direction of the effect (i.e., predicted to be negative, reflecting reduced accuracy for superordinate‐level trials).

#### 4.1.2 Computational model and evaluating performance

All modeling and evaluation procedures used in this study were the same as in Study 1. The counterbalanced design of the human experiment required us to present participants with some target object concepts that were not present in both components of the model (e.g., *rowboat* as a type of boat, and *dachshund* as a type of dog, were both absent from the linguistic component). There were 15 such objects, with membership spread across five of the eight basic‐level categories. These items were, therefore, excluded from the simulation according to the same criteria as Simulation 1 (*N* =  180 items: 30 true and 150 false). As a result, there was a total of 780 items included in the simulation (130 true, 650 false). Split by taxonomic level, there were 520 basic‐level items (65 true, 455 false) and 260 superordinate‐level items (65 true, 195 false).

### 4.2 Results

#### 4.2.1 Human data

As preregistered, we removed 17 trials from the analysis of accuracy (0.12% of all 14,400 responses) that had response times below 200 ms, indicating motor error.

Bayesian model comparisons showed overwhelming evidence for models containing a fixed effect of taxonomic level over a null model containing only random effects of participant and object/image BF_10_ =  3.69×10^37^). Participants were less likely to categorize an object correctly if it followed a superordinate‐level category label compared to a basic‐level category label [unstandardized *b* =  −1.20, 95% CI ± 0.17, *z* =  −13.79]. That is, as predicted, people's categorization behavior in a go/no‐go task exhibited the classic basic‐level advantage.

#### 4.2.2 Model evaluation

**Fig. 5:**
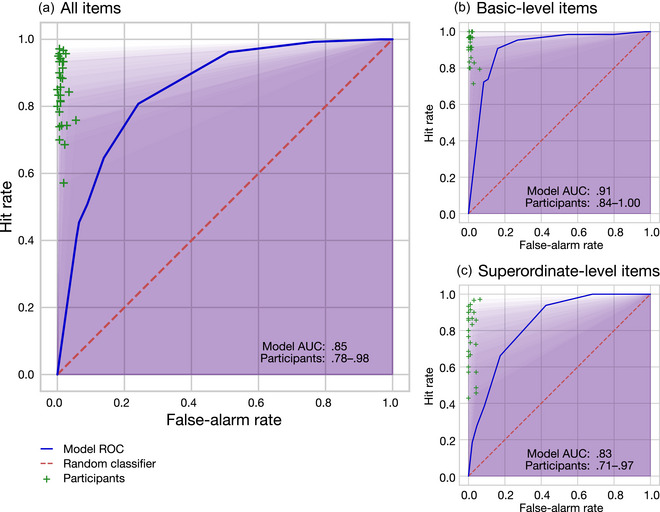
ROC curves of Simulation 2 category verification performance of model versus human participants for all items (Panel A), basic‐level items (Panel B), and superordinate‐level items (Panel C). Model ROC curve (solid blue line) is plotted across decision thresholds 0.0–1.0 compared to chance performance of a random classifier (dotted red line). Performance of each individual participant is indicated by a cross, with the area under the curve (AUC) shown as a shaded background region. Inset numbers per panel report AUC for the model and the range of AUC values for human participants.

Using the ROC‐AUC measure, the model again successfully performed category verification with AUC = .85, which was better than in Simulation 1. This level of performance fell well within the participant AUC range [.78, .98], which indicates that the model was about as good as human participants at categorizing the present item set. The all‐item ROC curves for model and participants are shown in Fig. 5A.

As predicted, and replicating Simulation 1, the model exhibited the basic‐level‐advantage effect: model AUC = .91 on basic‐level items and fell to AUC = .83 on superordinate‐level items. At both taxonomic levels, these model AUCs were comfortably within the respective participant ranges (basic [.84, 1.00], superordinate [.71, .97]); see Fig. 5B,C. Indeed, the magnitude of the model's basic‐level advantage (AUC difference = .09) was close to that of human participants (mean AUC difference = .07). These results indicate that the model could categorize at both the basic and superordinate level approximately as well as a random participant could, and that both the model and people found superordinate categorization more difficult than basic‐level categorization.

**Fig. 6:**
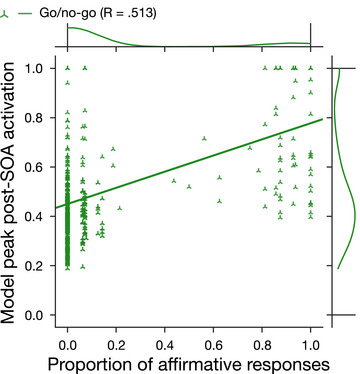
Scatterplot of peak activation level attained by each object concept within the model against the proportion of human participants who responded “yes” in Simulation 2 (i.e., accepted that object as a category member in a go/no‐go design). Marginal plots depict distribution densities. *R*‐value relates to linear trendline.

Lastly, and again as expected, the peak activation level reached by target object concepts in the model correlated with the likelihood of participants responding “yes” (i.e., *go* in the go/no‐go verification task: *r* = .51, log BF_+0_ =  116.29). That is, the more strongly a category label could boost the model's activation of an object concept (e.g., *bird* → *eagle*, or *container → lunchbox*), the more likely people were to accept that particular object as a category member (Fig. 6).

### 4.3 Discussion

This second, validation simulation corroborates the findings of Simulation 1. By using a fresh set of items and new participant data, we demonstrated that the model was not overfit to the original item set and that the findings replicate. Specifically, the linguistic‐sensorimotor model can perform category verification at a level similar to human participants at both basic and superordinate levels. Moreover, the model again produced an emergent basic‐level advantage, solely on the basic of spreading activation from the category label across linguistic‐distributional and sensorimotor components, without any explicit knowledge of taxonomic hierarchies. The human dataset in this study used a revised experimental paradigm which more closely aligned to the decision procedure undertaken by the model (i.e., go/no‐go rather than 2AFC decision task), and indeed the model ROC‐AUCs were themselves closer to the participant ROC‐AUCs than what we observed in Simulation 1. In addition, peak object activation in the model was strongly correlated with participants’ likelihood of accepting an object as a category member, even to the point of replicating common participant errors (e.g., both model and people were reluctant to accept a *yacht* as a member of the category *vehicle*). In this way, the model results support van Hoef et al. (2023) linguistic–sensorimotor preparation model of the basic‐level advantage in category verification.

Nonetheless, some differences still existed between model and human performance patterns. While AUCs were comparable, the shape of the ROC curves differed. Participants tended to have reasonably high hit‐rates and very low false‐alarm rates across items as a whole, while the model tended to allow more false alarms relative to hits at any given decision threshold (see Fig. 5). That is, people were generally conservative about pressing the “yes” button in the go/no‐no task, meaning they often missed true category members but also admitted a few false members. For instance, participants only produced false alarms to 1.3% of nonmembers, compared to a liberal 15–17% rate in the yes/no task featured in Simulation 1 (see Table S3 for more details). The unequal ratio of true:false items was not responsible for this conservative responding; we conducted a follow‐up study which balanced the numbers of true/false items each participant saw in the go/no‐go paradigm and it made little difference to results. Specifically, the conservative responding persisted (hit‐rates remained high and false alarms remained low at 2.0%: see Table S3) and the basic‐level advantage still emerged.^11^ We, therefore, conclude that the model's category verification behavior and emergent basic‐level advantage closely resembles that of human participants but has a more liberal decision bias (i.e., overall more likely to say “yes”) than participants.

## 5 Linguistic‐only and sensorimotor‐only models

According to the sensorimotor–linguistic preparation model of van Hoef et al. (2023), the basic‐level advantage emerges from the tendency of specific concepts (e.g., *Labrador*) to share more linguistic and/or sensorimotor experience with their basic‐level category concepts (e.g., *dog*) than with their superordinate‐level category concepts (e.g., *animal*). In this sense, while both linguistic and sensorimotor information contribute to the categorization process, the basic‐level advantage for a given item (or set of items) may be the result of the category → member relationship in linguistic terms, in sensorimotor terms, or both. Since the model in Simulations 1 and 2 comprised interacting linguistic and sensorimotor components, it remains unclear how each source of information contributes to model performance. We therefore investigated the separate contributions of linguistic and sensorimotor information by creating two modified versions of the model which contained only a linguistic or sensorimotor component and resimulated the data. We summarize here the main findings; full details are available in Supplementary Materials.

Results show that both linguistic‐distributional and sensorimotor information contribute to categorization results. Overall, in the full item sets from Simulations 1 and 2, removing linguistic information reduced the performance of the model, though it still remained above the level of chance (i.e., 0.50): see Table 1 and ROC‐AUC plots in the Appendix. That is, one can remove either component and still get better‐than‐chance categorization performance that in some cases lies within the human range. The Simulation 2 item set, with its systematic rotation of concepts across categories, shows that neither component alone performs as well as the full model where linguistic and sensorimotor information interacts. Critically, the basic‐level advantage emerged in both the sensorimotor‐only model (Simulation 1 item set) and the linguistic‐only model (Simulation 1 and 2 item sets). Indeed, looking at the magnitude of the simulated basic‐level advantage (i.e., AUC difference between basic and superordinate categorization), it appears that the model's basic‐level advantage was primarily driven by the sensorimotor component in Simulation 1 and by the linguistic component in Simulation 2.

**Table 1:** ROC‐AUC values for item sets from Simulations 1 and 2, for the sensorimotor‐only and linguistic‐only models, with the full model and participant ranges included for reference

ROC‐AUC values	Sensorimotor‐only model	Linguistic‐only model	Full model	Participant range
Simulation 1 item set				
All items	0.62	0.72	0.68	0.74–0.98
Basic‐level items	0.74	0.79	0.80	0.68–1.00
Superordinate‐level items	0.54	0.71	0.63	0.68–0.98
Simulation 2 item set				
All items	0.71	0.82	0.85	0.78–0.98
Basic‐level items	0.71	0.86	0.91	0.84–1.00
Superordinate‐level items	0.72	0.78	0.83	0.71–0.97

We speculate that the slightly different sensorimotor versus linguistic patterns across item sets is a function of the individual items involved, particularly superordinate items. In Simulation 1's item set, the difference between members and nonmembers (i.e., true and false items) appears to be unusually stark in linguistic terms because the linguistic‐only model achieved very good categorization performance on those items, but relatively small in sensorimotor terms because the sensorimotor‐only model performed only slightly better than chance. However, when we took a more systematic approach to rotating superordinate categories across nonmember concepts in Simulation 2's item set (i.e., increasing the range of false items), the pattern disappears. Indeed, in the Simulation 2 item set, performance on superordinate items in the sensorimotor‐only model is as good as at the basic level. This pattern of the results is consistent with the notion that concepts are easier to categorize when there is an overlap in sensorimotor experience and/or linguistic distributional knowledge between category and member concepts, and the basic‐level advantage emerges as an overall behavioral artifact of this linguistic and sensorimotor overlap.

## 6 General discussion

We have presented a computational model which simulates the label → picture category‐verification task using a combination of linguistic distributional and sensorimotor information to spread activation from the category name (e.g., *dog* or *animal*) to the target object concept (e.g., *Labrador*). In simulating the task, the peak activation of targets in the model strongly predicted the likelihood that participants would accept the item as a category member. Critically, despite having no in‐built knowledge of a hierarchical taxonomy of concepts, the model also reproduced the phenomenon of the basic‐level advantage, with more hits and fewer false‐positives on decisions for basic‐level categories than for more generalized superordinate categories.

The empirical effect of the basic‐level advantage in categorization is well established. Traditional accounts of conceptual processing assume that the phenomenon arises from the fact that basic‐level categories hold some elevated status in semantic memory: either that they are privileged entry‐points in a hierarchical taxonomy (e.g., Jolicoeur et al., 1984), or that they are simply and maximally differentiable by the semantic features of member concepts (e.g., Murphy & Brownell, 1985). Our computational model of spreading activation in semantic memory, on the other hand, incorporates neither a hierarchical arrangement of concepts, nor a database of semantic features, simply a large‐scale mesh of connections between concepts based on linguistic and sensorimotor experience. Nevertheless, in simulating the category‐verification task, it still exhibits the basic‐level advantage. The present results, therefore, support an alternative proposal of a linguistic–simulation preparation model (van Hoef et al., 2023), where the basic‐level advantage arises naturally from how category concepts (e.g., basic‐level *dog*, or more generalized *animal*) share linguistic‐distributional and/or sensorimotor experience with specific object concepts (e.g., *Labrador*). When a participant (or the model) is presented with a category label (e.g., *dog*), it activates linguistic distributional knowledge about the label's contexts and a sensorimotor representation of the referent concept. Activation spreads from there, automatically, to other, related words and concepts. When a participant (or the model) is presented with the subsequent target object, it will be verified as a category member if its representation has been sufficiently preactivated by the category label, which occurs when there is sufficient overlap between the target object representation and the representations activated by the category label. Category verification succeeds because the sensorimotor and/or linguistic distributional profiles of taxonomically related concepts (e.g., *dog* and *Labrador*) generally overlap to a greater extent than the profiles of taxonomically unrelated concepts (e.g., *dog* and *daffodil*). Critically, the basic‐level advantage emerges because the sensorimotor–linguistic profile of an object concept will often—though not necessarily always—overlap more with that of a less‐generalized category concept (e.g., basic *dog* and *Labrador*) than a more‐generalized category concept (e.g., superordinate *animal* and *Labrador*).

Our model allows activation to spread indirectly between words and concepts (i.e., via intermediate neighbors), which Banks et al. (2021) found was critical to their simulation of category production behavior. Nonetheless, one might question whether indirect activations are necessary in category verification, particularly since van Hoef et al. (2023) found linguistic and sensorimotor effects using only *direct* relationships between category labels and target objects. For example, in our model, the category label *dog* can activate the target concept *Labrador* via a chain of nearby intermediates such as (in the linguistic component) *barking* and *puppy*, or it can activate *Labrador* directly if the distance is not so great that activation decays before it reaches it. In order to examine whether indirect activation was important to category verification, we impaired the model's ability to spread indirect activation within each component and tested how it affected model performance in Simulations 1 and 2 (see Supplementary Materials for full report). Results showed that, without indirect activations across each component, model performance was severely diminished (AUC dropped from .68 to .60 in Simulation 1, and from .85 to .60 in Simulation 2) and well below the level of human performance. Moreover, when we additionally prevented the model from spreading activation *between* linguistic and sensorimotor components (i.e., another form of indirect activation), it eliminated the model's ability to perform category verification (AUCs dropped to .44 and .50 for Simulation 1 and 2, respectively). That is, allowing conceptual activation to spread indirectly from word to word, from sensorimotor concept to concept, and from word to referent concept (and vice versa), is critical to the simulation of category verification in our model. Such a finding is consistent with linguistic–sensorimotor theories that assume conceptual processing involves interactive waves of activation between word labels and their sensorimotor referents (e.g., Barsalou et al., 2008; Connell & Lynott, 2014; Louwerse, 2018).

In category‐verification experiments, the basic‐level advantage emerges in response latencies (i.e., basic‐level judgments are faster than superordinate judgments) as well as in the eventual decision (e.g., Murphy & Brownell, 1985; Rosch et al., 1976; van Hoef et al., 2023). However, we did not set about to model latencies in the present paper because the nature of the label → picture category verification task means that latencies are strongly influenced by visual object recognition processes, which are not part of what our model captures. For instance, van Hoef et al. (2023) found that, although linguistic and sensorimotor information successfully predicted hit‐rate latencies, these predictors explained only a small proportion of variance in absolute terms (1.2–2.4%).^12^ By contrast, linguistic and sensorimotor information successfully predicted categorization accuracy with up to 12.1% of variance explained. Nonetheless, it would be interesting for future work to model the timecourse of categorical decisions in more detail. We believe that the current form of the model offers a plausible approximation of conceptual activation processes involved in a label → picture category verification task. However, since human latencies in this task are strongly influenced by factors other than conceptual activation processes (i.e., particularly regarding visual object recognition), more research is first needed to understand the nonrandom variance in categorization latency data for both hits and false alarms.

The model presented here, together with the model of Banks et al. (2021), is a general‐purpose framework of spreading conceptual activation across a full‐size conceptual system, that implements a theory of conceptual processing in which both linguistic and sensorimotor connections contribute, at different speeds and degrees, to participants’ behavior. Our framework can handle different tasks simply by adapting the front end (i.e., how stimuli are input to the model and how model outputs are interpreted as particular decisions or behaviors). In this way—by first formalizing a theory and then implementing the specification computationally—we create *task‐performing models* (Kriegeskorte & Douglas, 2018; Lake, Ullman, Tenenbaum, & Gershman, 2017; Newell, 1973): executable theories which generate their own precise predictions. Such computational models in effect simulate a hypothetical world in which the theory is true, demonstrating whether (or not) some phenomenon of interest arises (Borsboom et al., 2021). Insofar as the computational model simulates (reproduces) a phenomenon observed in participant's behavior, it has explanatory value. While the tasks simulated here and by Banks et al. (2021) are categorization tasks, the framework could equally be applied to other tasks which involve semantic activation. We are currently pursuing this research.

In summary, the present paper provides computational evidence for the linguistic–sensorimotor preparation model of the basic‐level advantage in category verification (van Hoef et al., 2023). Concepts are easier to categorize when there is a high degree of overlap in sensorimotor experience and/or linguistic‐distributional knowledge between category and member concepts, and the basic‐level advantage emerges as an overall behavioral artifact of this linguistic and sensorimotor overlap. Future work could usefully investigate how individual differences in linguistic and/or sensorimotor experience can mediate this emergent effect (e.g., expertise: Tanaka & Taylor, 1991). More broadly, these findings support the use of the linguistic shortcut (Connell, 2019) during categorization, whereby word labels and their distributional patterns provide a representational heuristic that enables more efficient conceptual processing than would otherwise be possible. That is, consistent with linguistic–sensorimotor theories (Barsalou et al., 2008; Connell & Lynott, 2014; Louwerse, 2011), the conceptual system is both grounded and linguistic in nature.

## Funding

This work is part of a project that has received funding from the European Research Council (ERC) under the European Union's Horizon 2020 research and innovation program (Grant agreement No. 682848) to LC.

## Conflicts of interest

We have no conflicts of interest to disclose.

All Supplementary Materials, data, and code are available at https://osf.io/m5hjb.

### Open Research Badges

This article has earned Open Data and Open Materials badges. Data and materials are available at https://osf.io/m5hjb.

## Figures and Tables

**Fig. A1 cogs70025-fig-0007:**
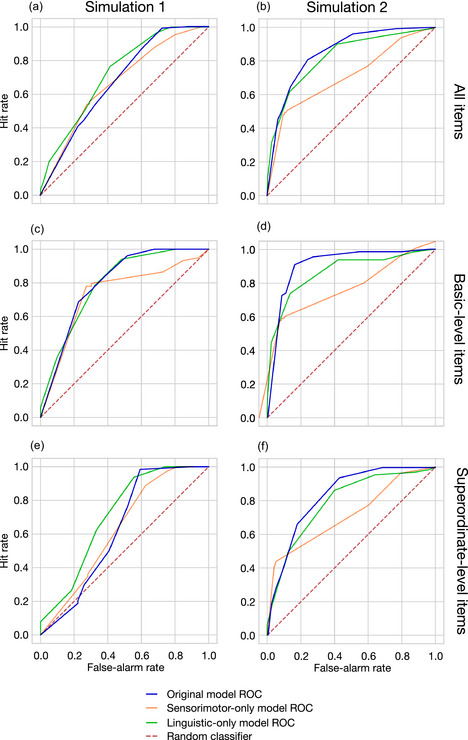
ROC curves for the original unaltered, sensorimotor‐only and linguistic‐only models. Left‐column panels (A, C, E) show results from Simulation 1's item set; right‐column panels (B, D, F) show results from Simulation 2's item set. First‐row panels (A, B) show results for all items; middle‐row panels (C, D) show results for basic‐level items only; bottom‐row panels (E, F) show results for superordinate‐level items only. ROC curves are plotted across decision thresholds 0.0–1.0 compared to chance performance of a random classifier (dotted red line).

## Data Availability

All materials, images, data, and code associated with this article are available on OSF at https://osf.io/m5hjb and licensed under a Creative Commons Attribution 4.0 International License (CC‐BY), which permits use, sharing, adaptation, distribution, and reproduction in any medium or format, as long as you give appropriate credit to the original authors and the source, provide a link to the Creative Commons license, and indicate if changes were made.

## Notes

1 Across different datasets, feature similarity was outperformed by sensorimotor distance and measures of either linguistic distributional similarity or taxonomic distance.

2 PPMI 6‐gram scores were found by Banks, Wingfield, and Connell (2021) to outperform more complex distributional models when predicting category production behavior, which is consistent with previous research showing that n‐gram language models can capture many semantic effects (Louwerse, 2011), particularly in tasks of medium‐to‐high semantic complexity (Wingfield & Connell 2022b).

3 Capacity of 3000 items is a rough approximation of the number of concepts in long‐term memory that may retain trace activation in an ongoing task, such as the linguistic words and sensorimotor referents involved in maintaining the plot of a novel in a situation model (e.g., Zwaan & Madden, 2005) or when asked to remember large sets of thousands of pictured objects (Brady, Konkle, Alvarez, & Oliva, 2008); see also Banks et al. (2021).

4 Minkowski‐3 distance is Minkowski distance with an exponent parameter of 3. It is a generalization of Euclidean distance (which equates to Minkowski distance with exponent 2) that places less weight on low‐magnitude dimensions, so the sensorimotor distance between two concepts is influenced most by the dimensions with strongest sensorimotor experience ratings. When measured from the origin, Lynott, Connell, Brysbaert, Brand, and Carney (2020) found that Minkowski‐3 distance outperformed other composite measures of sensorimotor strength in predicting semantic facilitation in word recognition.

5 The delay comprised a fixed number of ticks that elapsed before propagating cross‐component activation, based on the theoretical rationale that processing within a given representational format is less effortful than moving across different formats (e.g., Louwerse, 2018). The delay was fixed but asymmetric: 21 ticks in the linguistic → sensorimotor direction (i.e., time required for a word to activate its sensorimotor referent) and 56 ticks in the sensorimotor → linguistic direction (i.e., it takes longer to activate the name of a sensorimotor concept than for a word to activate its referent). See Simulation 1 for details of optimizing free parameters in the model and Supplementary Materials for a list of all model parameter settings and sources.

6 As a necessary expediency to limit computational resource requirements, newly activated items would not themselves propagate further activation during the target stage. Therefore, in the target stage, the activation level of a given linguistic node or sensorimotor concept point is only affected by activation that was already initiated in the cue stage (i.e., spread from the category label) and/or by activation incrementally applied to the object concept during the target stage itself.

7 Of the original 144 true items extracted from van Hoef et al.’s dataset, we excluded 13 items where the category word was repeated in the object label (e.g., *table* → *coffee table*), which would have given the model an unfair advantage by directly preactivating the object label, and a further 16 items that specified concepts not available in both components of the model (e.g., the object label *switchblade* was absent from the linguistic component), which would have artificially penalized the model by impeding initial propagation of activation. Similarly, of the original 65 false items, we excluded seven that specified concepts not available in both components of the model.

8 We also examined the extent to which our chosen SOA duration was responsible for model performance. We reran the analysis with different SOA durations in the model, and found performance across all items to be stable from approximately 200 ticks. Notably, performance for basic‐level items was good across all SOA, while performance of superordinate items started poor and peaked around our chosen SOA of 241 ticks. While we were not explicitly modeling the timecourse of category verification, this distinction is nonetheless consistent with how the basic‐level advantage manifests in response times (i.e., superordinate categorization takes longer than basic‐level categorization). Full details are in Supplementary Materials.

9 Settings for free parameters were optimized with reference only to human Dataset 1, with Dataset 2 not examined until after model parameters were fixed. In practice, both datasets were very similar to one another, which is unsurprising given that they were replications of the same item set across different participant samples. Thus, we were optimizing for maximum ROC‐AUC for the model on the *items* used, meaning that similar model performance across Datasets 1 and 2 cannot provide evidence against overfitting, for which a different item set is required.

10 We also ran a follow‐up study that balanced the numbers of true/false items each participant saw in the go/no‐go paradigm: see Discussion for findings and Supplementary Materials for a full report.

11 Bayesian model comparisons showed overwhelming evidence for the effect of taxonomic level over a null model containing only random effects of participant and object/image (BF_10_ =  1.36×10^22^), where participants were less likely to categorize an object correctly if it followed a superordinate category label compared to a basic‐level label [unstandardized *b* =  −0.90, 95% CI ± .17, *z* =  −10.55, *p* < .001]. Taking model performance from Simulation 2 (i.e., the item set was identical) and comparing it to human performance in this balanced no/no‐go task, basic‐level AUC of .91 was just below the participant range [.92, 1.00] and superordinate level AUC of .83 was well within the participant range [.75, .99]. A full report is available in Supplementary Materials.

12 This point applies to any predictors relating to semantic memory structure: competing predictors such as taxonomic levels or typicality performed similarly or worse.

## 1

 [Fig cogs70025-fig-0007]

## References

[cogs70025-bib-0001] Anwyl‐Irvine, A. L. , Massonnié, J. , Flitton, A. , Kirkham, N. , & Evershed, J. K. (2020). Gorilla in our midst: An online behavioral experiment builder. Behavior Research Methods, 52(1), 388–407. 10.3758/s13428-019-01237-x 31016684 PMC7005094

[cogs70025-bib-0085] Baddeley, A. (2000). The episodic buffer: A new component of working memory?. Trends in cognitive sciences, 4(11), 417–423. 10.1016/S1364-6613(00)01538-2 11058819

[cogs70025-bib-0002] Barsalou, L. W. (1999). Perceptual symbol systems. Behavioral and Brain Sciences, 22(4), 577–660. 10.1017/S0140525x99002149 11301525

[cogs70025-bib-0003] Barsalou, L. W. , Niedenthal, P. M. , Barbey, A. , & Ruppert, J. (2003). Social embodiment. In B. Ross (Ed.), The psychology of learning and motivation (Vol. 43, pp. 43–92). Academic Press.

[cogs70025-bib-0004] Barsalou, L. W. , Santos, A. , Simmons, W. K. , & Wilson, C. D. (2008). Language and simulation in conceptual processing. In M. De Vega , A. M. Glenberg , & A. C. Graesser (Eds.), Symbols and embodiment: Debates on meaning and cognition (pp. 245–283). Oxford University Press.

[cogs70025-bib-0005] Banks , B. , & Connell, L. (2022a). Category production norms for 117 concrete and abstract categories. Behavior Research Methods. 55, 1292–1313. 10.3758/s13428-021-01787-z 35650380 PMC10126059

[cogs70025-bib-0006] Banks, B. , & Connell, L. (2022b). Multi‐dimensional sensorimotor grounding of concrete and abstract categories. Philosophical Transactions of the Royal Society B: Biological Sciences, 378(1870), Article 20210366. 10.1098/rstb.2021.0366 PMC9791484 36571121

[cogs70025-bib-0007] Banks, B. , & Connell, L. (2024). Access to inner language enhances memory for events. Journal of Experimental Psychology: Learning, Memory, and Cognition, 50(10), 1592–1615. 10.1037/xlm0001351 38934927

[cogs70025-bib-0008] Banks, B. , Wingfield, C. , & Connell, L. (2021). Linguistic distributional knowledge and sensorimotor grounding both contribute to semantic category production. Cognitive Science, 45(10), e13055. 10.1111/cogs.13055 34647346

[cogs70025-bib-0082] Binder, J. R. , Conant, L. L. , Humphries, C. J. , Fernandino, L. , Simons, S. B. , Aguilar, M. , & Desai, R. H. (2016). Toward a brain‐based componential semantic representation. Cognitive Neuropsychology, 33(3–4), 130–174. 10.1080/02643294.2016.1147426 27310469

[cogs70025-bib-0009] Bird, S. , Klein, E. , & Loper, E. (2009). Natural language processing with Python. O'Reilly Media.

[cogs70025-bib-0010] Borsboom, D. , van der Maas, H. L. J. , Dalege, J. , Kievit, R. A. , & Haig, B. D. (2021). Theory construction methodology: A practical framework for building theories in psychology. Perspectives on Psychological Science, 16(4), 756–766. 10.1177/1745691620969647 33593167

[cogs70025-bib-0011] Brady, T. F. , Konkle, T. , Alvarez, G. A. , & Oliva, A. (2008). Visual long‐term memory has a massive storage capacity for object details. Proceedings of the National Academy of Sciences, 105(38), 14325–14329. 10.1073/pnas.0803390105 PMC2533687 18787113

[cogs70025-bib-0012] Brown, R. (1958). How shall a thing be called? Psychological Review, 65(1), 14–21. 10.1037/h0041727 13505978

[cogs70025-bib-0013] Brysbaert, M. , Mandera, P. , McCormick, S. F. , & Keuleers, E. (2019). Word prevalence norms for 62,000 English lemmas. Behavior Research Methods, 51(2), 467–479. 10.3758/s13428-018-1077-9 29967979

[cogs70025-bib-0014] Brysbaert, M. , Warriner, A. B. , & Kuperman, V. (2014). Concreteness ratings for 40 thousand generally known English word lemmas. Behavior Research Methods, 46, 904–911. 10.3758/s13428-013-0403-5 24142837

[cogs70025-bib-0015] Bullinaria, J. A. , & Levy, J. P. (2007). Extracting semantic representations from word co‐occurrence statistics: A computational study. Behavior Research Methods, 39(3), 510–526. 10.3758/BF03193020 17958162

[cogs70025-bib-0016] Connell, L. (2019). What have labels ever done for us? The linguistic shortcut in conceptual processing. Language, Cognition and Neuroscience, 3798, 1308–1318. 10.1080/23273798.2018.1471512

[cogs70025-bib-0017] Connell, L. , & Lynott, D. (2012). Strength of perceptual experience predicts word processing performance better than concreteness or imageability. Cognition, 125, 452–465. 10.1016/j.cognition.2012.07.010 22935248

[cogs70025-bib-0018] Connell, L. , & Lynott, D. (2013). Flexible and fast: Linguistic shortcut affects both shallow and deep conceptual processing. Psychonomic Bulletin & Review, 20(3), 542–550. 10.3758/s13423-012-0368-x 23307559

[cogs70025-bib-0019] Connell, L. , & Lynott, D. (2014). Principles of representation: Why you can't represent the same concept twice. Topics in Cognitive Science, 6(3), 390–406. 10.1111/tops.12097 24895329

[cogs70025-bib-0020] Connell, L. , Lynott, D. , & Banks, B. (2018). Interoception: The forgotten modality in perceptual grounding of abstract and concrete concepts. Philosophical Transactions of the Royal Society B: Biological Sciences, 373(1752), Article 20170143. 10.1098/rstb.2017.0143 PMC6015840 29915011

[cogs70025-bib-0021] Cooper, R. P. , & Guest, O. (2014). Implementations are not specifications: Specification, replication and experimentation in computational cognitive modeling. Cognitive Systems Research, 27, 42–49. 10.1016/j.cogsys.2013.05.001

[cogs70025-bib-0022] Davis, C. P. , & Yee, E. (2021). Building semantic memory from embodied and distributional language experience. Wiley Interdisciplinary Reviews: Cognitive Science, 12(5), e1555. 10.1002/wcs.1555 33533205

[cogs70025-bib-0023] Dymarska, A. , Connell, L. , & Banks, B. (2022). Linguistic bootstrapping allows more real‐world object concepts to be held in mind. Collabra: Psychology, 8(1), 40171. 10.1525/collabra.40171

[cogs70025-bib-0024] Fawcett, T. (2006). An introduction to ROC analysis. Pattern Recognition Letters, 27(8), 861–874. 10.1016/j.patrec.2005.10.010

[cogs70025-bib-0025] Ferreira, F. , Bailey, K. G. , & Ferraro, V. (2002). Good‐enough representations in language comprehension. Current Directions in Psychological Science, 11, 11–15. 10.1111/1467-8721.00158

[cogs70025-bib-0026] Fodor, J. A. (1975). The language of thought. Harvard University Press.

[cogs70025-bib-0027] Guest, O. , & Martin, A. E. (2021). How computational modeling can force theory building in psychological science. Perspectives on Psychological Science, 16(4), 789–802. 10.1177/1745691620970585 33482070

[cogs70025-bib-0028] Hanley, J. A. , & McNeil, B. J. (1982). The meaning and use of the area under a receiver operating characteristic (ROC) curve. Radiology, 143(1), 29–36. 10.1148/radiology.143.1.7063747 7063747

[cogs70025-bib-0080] Harnad, S. (2005). To cognize is to categorize: Cognition is categorization. In H. Cohen & C. Lefebvre (Eds.), Handbook of Categorization in Cognitive Science (pp, 20–45). Elsevier.

[cogs70025-bib-0029] JASP Team . (2022). JASP (Version 0.16.3) [Computer software].

[cogs70025-bib-0030] Jiang, L. , Stocco, A. , Losey, D. M. , Abernethy, J. A. , Prat, C. S. , & Rao, R. P. (2019). BrainNet: A multi‐person brain‐to‐brain interface for direct collaboration between brains. Scientific Reports, 9(1), 1–11. 10.1038/s41598-019-41895-7 30992474 PMC6467884

[cogs70025-bib-0031] Jolicoeur, P. , Gluck, M. , & Kosslyn, S. (1984). Pictures and names: Making the connection. Cognitive Psychology, 16(2), 243–275. 10.1016/0010-0285(84)90009-4 6734136

[cogs70025-bib-0032] Johnson, K. E. , & Mervis, C. B. (1997). Effects of varying levels of expertise on the basic level of categorization. Journal of Experimental Psychology: General, 126(3), 248–277. 10.1037/0096-3445.126.3.248 9281832

[cogs70025-bib-0033] Kriegeskorte, N. , & Douglas, P. K. (2018). Cognitive computational neuroscience. Nature Neuroscience, 21(9), 1148–1160. 10.1038/s41593-018-0210-5 30127428 PMC6706072

[cogs70025-bib-0034] Lake, B. M. , Ullman, T. D. , Tenenbaum, J. B. , & Gershman, S. J. (2017). Building machines that learn and think like people. Behavioral and Brain Sciences, 40, E253. 10.1017/S0140525x16001837 27881212

[cogs70025-bib-0035] Louwerse, M. M. (2011). Symbol interdependency in symbolic and embodied cognition. Topics in Cognitive Science, 3(2), 273–302. 10.1111/j.1756-8765.2010.01106.x 25164297

[cogs70025-bib-0036] Louwerse, M. M. (2018). Knowing the meaning of a word by the linguistic and perceptual company it keeps. Topics in Cognitive Science, 10(3), 573–589. 10.1111/tops.12349 29851286

[cogs70025-bib-0037] Louwerse, M. , & Connell, L. (2011). A taste of words: Linguistic context and perceptual simulation predict the modality of words. Cognitive Science, 35(2), 381–398. 10.1111/j.1551-6709.2010.01157.x 21429005

[cogs70025-bib-0083] Louwerse, M. , & Jeuniaux, P. (2008). Language comprehension is both embodied and symbolic. In M. De Vega , A. Glenberg , & A. C. Graesser (Eds.), Symbols and Embodiment: Debates on meaning and cognition (pp. 309–426). Oxford, England: Oxford University Press. 10.1093/acprof:oSo/9780199217274.003.0015

[cogs70025-bib-0038] Louwerse, M. M. , & Jeuniaux, L. (2010). The linguistic and embodied nature of conceptual processing. Cognition, 114(1), 96–104. 10.1016/j.cognition.2009.09.002 19818435

[cogs70025-bib-0039] Lynott, D. , & Connell, L. (2010). Embodied conceptual combination. Frontiers in Psychology, 1(216), 1–14. 10.3389/fpsyg.2010.00212 21833267 PMC3153817

[cogs70025-bib-0040] Lynott, D. , Connell, L. , Brysbaert, M. , Brand, J. , & Carney, J. (2020). The Lancaster Sensorimotor Norms: Multidimensional measures of perceptual and action strength for 40,000 English words. Behavior Research Methods, 52(3), 1271–1291. 10.3758/s13428-019-01316-z 31832879 PMC7280349

[cogs70025-bib-0041] Mani, N. , & Plunkett, K. (2010). In the infant's mind's ear: Evidence for implicit naming in 18‐month‐olds. Psychological Science, 21(7), 908–913. 10.1177/09567976103733 20519485

[cogs70025-bib-0042] Medin, D. L., & Schaffer, M. M. (1978). Context theory of classification learning. Psychological Review, 85(3), 207–238. 10.1037/033-295X.85.3.207

[cogs70025-bib-0043] Medin, D. L. , & Shoben, E. J. (1988). Context and structure in conceptual combination. Cognitive Psychology, 20(2), 158–190. 10.1016/0010-0285(88)90018-7 3365938

[cogs70025-bib-0044] Metz, C. E. (1978) Basic principles of ROC analysis. In L. M. Freeman & M. D. Blaufox (Eds.) Seminars in nuclear medicine (Vol. 8, pp. 283–298) No. 4, WB Saunders. 10.1016/S0001-2998(78)80014-2 112681

[cogs70025-bib-0045] Meyer, A. S. , Belke, E. , Telling, A. L. , & Humphreys, G. W. (2007). Early activation of object names in visual search. Psychonomic Bulletin & Review, 14, 710–716. 10.3758/BF03196826 17972738

[cogs70025-bib-0046] Minda, J. P. , & Smith, J. D. (2001). Prototypes in category learning: The effects of category size, category structure and stimulus complexity. Journal of Experimental Psychology: Learning, Memory and Cognition, 27(3), 775–779. 10.1037/0278-7393.27.3.775 11394680

[cogs70025-bib-0047] Mueller, S. T. , & Krawitz, A. (2009). Reconsidering the two‐second decay hypothesis in verbal working memory. Journal of Mathematical Psychology, 53(1), 14–25. 10.1016/j.jmp.2008.11.001

[cogs70025-bib-0048] Murphy, G. L. (2016). Explaining the basic‐level concept advantage in infants…or is it the superordinate‐level advantage? In B. H. Ross (Ed.), Psychology of learning and motivation (Vol. 64, pp. 57–92). Academic Press. 10.1016/bs.plm.2015.09.002

[cogs70025-bib-0049] Murphy, G. L. , & Brownell, H. H. (1985). Category differentiation in object recognition: Typicality constraints on the basic category advantage. Journal of Experimental Psychology: Learning, Memory, and Cognition, 11(1), 70–84. 10.1037/0278-7393.11.1.70 3156953

[cogs70025-bib-0050] Murphy, G. L. , & Smith, E. E. (1982). Basic‐level superiority in picture categorization. Journal of Verbal Learning and Verbal Behavior, 21(1), 1–20. 10.1016/S0022-5371(82)90412-1

[cogs70025-bib-0051] Newell, A. (1973). You can't play 20 Questions with nature and win: Projective comments on the papers of this symposium. In W. G. Chase (Ed.), Visual information processing (pp. 283–308). Academic Press. 10.1016/B978-0-12-170150-5.50012-3

[cogs70025-bib-0052] Nosofsky, R. M. (1986). Attention, similarity, and the identification‐categorization relationship. Journal of Experimental Psychology: General, 115(1), 39–57. 10.1037/0096-3445.115.1.39 2937873

[cogs70025-bib-0084] Nosofsky, R. M. (1987). Attention and learning processes in the identification and categorization of integral stimuli. Journal of Experimental Psychology: Learning, Memory and Cognition, 13, 87–108. 2949055 10.1037//0278-7393.13.1.87

[cogs70025-bib-0053] Nosofsky, R. M. , & Palmeri, T. J. (1997). An exemplar‐based random walk model of speeded classification. Psychological Review, 104(2), 266–300. 10.1037/0033-295X.104.2.266 9127583

[cogs70025-bib-0054] Pothos, E. M., & Chater, N. (2002). A simplicity principle in unsupervised human categorization. Cognitive Science, 26(3), 303–343. 10.1207/s15516709cog2603_6

[cogs70025-bib-0055] Quinn, P. C. , & Johnson, M. H. (1997). The emergence of perceptual category representations in young infants: A connectionist analysis. Journal of Experimental Child Psychology, 66(2), 236–263. 10.1006/jecp.1997.2385 9245477

[cogs70025-bib-0056] Quinn, P. C. , & Johnson, M. H. (2000). Global‐before‐basic object categorization in connectionist networks and 2‐month‐old infants. Infancy, 1(1), 31–46. 10.1207/S15327078IN0101_04 32680309

[cogs70025-bib-0057] Reilly, J. , Shain, C. , Valentina, B. , Kuhnke, P. , Vigliocco, G. , Peelle, J.E. , … Vinson, D. (2024). What we mean when we say semantic: Towards a multidisciplinary semantic glossary. Psychonometric Bulletin & Review, 10.3758/s13423-024-02556-7 PMC11836185 39231896

[cogs70025-bib-0058] Riordan, B. , & Jones, M. N. (2011). Redundancy in perceptual and linguistic experience: Comparing feature‐based and distributional models of semantic representation. Topics in Cognitive Science, 3(2), 303–345. 10.1111/j.1756-8765.2010.01111.x 25164298

[cogs70025-bib-0059] Rogers, T. T. , & McClelland, J. L. (2004). Semantic cognition: A parallel distributed processing approach. MIT Press. 10.7551/mitpress/6161.001.0001 12671647

[cogs70025-bib-0060] Rosch, E. (1978). Principles of categorization. In E. Rosch & B. Lloyd (Eds.), Cognition & categorization. Lawrence Erlbaum Associates.

[cogs70025-bib-0061] Rosch, E. , Mervis, C. B. , Gray, W. , Johnson, D. , & Boyes‐Braem, P. (1976). Basic objects in natural categories. Cognitive Psychology, 8(3), 382–439. 10.1016/0010-0285(76)90013-X

[cogs70025-bib-0062] Rosch, E. , & Mervis, C. B. (1975). Family resemblances: Studies in the internal structure of categories. Cognitive Psychology, 7(4), 573–605. 10.1016/0010-0285(75)90024-9

[cogs70025-bib-0063] Santos, A. , Chaigneau, S. E. , Simmons, W. K. , & Barsalou, L. W. (2011). Property generation reflects word association and situated simulation. Language and Cognition, 3(1), 83–119. 10.1515/langcog.2011.004

[cogs70025-bib-0064] Schönbrodt, F. D. , Wagenmakers, E.‐J. , Zehetleitner, M. , & Perugini, M. (2017). Sequential hypothesis testing with Bayes factors: Efficiently testing mean differences. Psychological Methods, 22(2), 322–339. 10.1037/met0000061 26651986

[cogs70025-bib-0065] Schweickert, R. , & Boruff, B. (1986). Short‐term memory capacity: Magic number or magic spell? Journal of Experimental Psychology: Learning, Memory, and Cognition, 12(3), 419–425. 10.1037/0278-7393.12.3.419 2942626

[cogs70025-bib-0066] Smith, J. D. , & Minda, J. P. (2000). Thirty categorization results in search of a model. Journal of Experimental Psychology: Learning, Memory and Cognition, 26(1), 3–27. 10.1037/0278-7393.26.1.3 10682288

[cogs70025-bib-0067] Tanaka, J. , & Taylor, M. (1991). Object categories and expertise: Is the basic level in the eye of the beholder? Cognitive Psychology, 23(3), 457–482. 10.1016/0010-0285(91)90016-H

[cogs70025-bib-0068] van Heuven, W. J. B. , Mandera, P. , Keuleers, E. , & Brysbaert, M. (2014). Subtlex‐UK: A new and improved word frequency database for British English. Quarterly Journal of Experimental Psychology, 67(6), 1176–1190. 10.1080/17470218.2013.850521 24417251

[cogs70025-bib-0069] van Hoef, R. , Connell, L. , & Lynott, D. (2023). The effects of sensorimotor and linguistic information on the basic‐level advantage. Cognition, 241, Article 105606. 10.1016/j.cognition.2023.105606 37722237

[cogs70025-bib-0070] van Hoef, R. , Lynott, D. , & Connell, L. (2024). Timed picture naming norms for 800 photographs of 200 objects in English. Behaviour Research Methods, 56, 6655–6672. 10.3758/s13428-024-02380-w PMC11362483 38504079

[cogs70025-bib-0071] Vigliocco, G. , Meteyard, L. , Andrews, M. , & Kousta, S. (2009). Toward a theory of semantic representation. Language and Cognition, 1(2), 219–247. 10.1515/LANGCOG.2009.011

[cogs70025-bib-0072] Villani, C. , Lugli, L. , Liuzza, M. T. , & Borghi, A. M. (2019). Varieties of abstract concepts and their multiple dimensions. Language and Cognition, 11(3), 403–430. 10.1017/langcog.2019.23

[cogs70025-bib-0073] Wagenmakers, E. J. (2007). A practical solution to the pervasive problems of *p* values. Psychonomic Bulletin & Review, 14(5), 779–804. 10.3758/BF03194105 18087943

[cogs70025-bib-0074] Waskom, M. (2021). seaborn: Statistical data visualization. Journal of Open Source Software, 6(60), 3021. 10.21105/joss.03021

[cogs70025-bib-0075] Wingfield, C. , & Connell, L. (2022a). Sensorimotor distance: A grounded measure of semantic similarity for 800 million concept pairs. Behavior Research Methods. 10.3758/s13428-022-01965-7 PMC10615916 36131199

[cogs70025-bib-0076] Wingfield, C. , & Connell, L. (2022b). Understanding the role of linguistic distributional knowledge in cognition. Language, Cognition and Neuroscience, 37(10), 1220–1270. 10.1080/23273798.2022.2069278

[cogs70025-bib-0077] Wittgenstein, L. (1953). Philosophical investigations. Blackwell.

[cogs70025-bib-0081] Yee, E. , Chrysikou, E. G. , Hoffman, E. , & Thompson‐Schill, S. L. (2013). Manual Experience Shapes Object Representations. Psychological Science, 24(6), 909–919. 10.1177/0956797612464658 23633520 PMC4138717

[cogs70025-bib-0078] Zwaan, R. A. (2004). The immersed experience: Toward an embodied theory of language comprehension. In B. H. Ross (Ed.), The psychology of language and motivation (Vol. 44). Academic Press.

[cogs70025-bib-0079] Zwaan, R. A. , & Madden, C. J. (2005). Embodied sentence comprehension. In D. Pecher & R. A. Zwaan (Eds.), Grounding cognition: The role of action and perception in memory, language, and thinking (pp. 224–245). Cambridge University Press.

